# Antibiotic Resistance of Uropathogens Isolated from Patients Hospitalized in District Hospital in Central Poland in 2020

**DOI:** 10.3390/antibiotics10040447

**Published:** 2021-04-16

**Authors:** Barbara Kot, Agata Grużewska, Piotr Szweda, Jolanta Wicha, Urszula Parulska

**Affiliations:** 1 Institute of Biological Sciences, Faculty of Exact and Natural Sciences, Siedlce University of Natural Sciences and Humanities, 14 Bolesława Prusa Str., 08-110 Siedlce, Poland; ulapar91@gmail.com; 2 Institute of Agriculture and Horticulture, Faculty of Agrobioengineering and Animal Husbandry, Siedlce University of Natural Sciences and Humanities, 12 Bolesława Prusa Str., 08-110 Siedlce, Poland; agata.gruzewska@uph.edu.pl; 3 Department of Pharmaceutical Technology and Biochemistry, Faculty of Chemistry, Gdańsk University of Technology, 11/12 G. Narutowicza Str., 80-233 Gdańsk, Poland; piotr.szweda@pg.edu.pl; 4 Medical Microbiological Laboratory, Our Lady of Perpetual Help Hospital, 1/3 Gdyńska Str., 05-200 Wołomin, Poland; jolawich@wp.pl

**Keywords:** urinary tract infection, uropathogenes, resistance to antibiotics, *Escherichia coli*, *Klebsiella pneumoniae*, *Proteus mirabilis*, *Enterococcus faecium*

## Abstract

The aim of this study was to determine antibiotic resistance patterns and the prevalence of uropathogenes causing urinary tract infections (UTIs) in patients hospitalized in January–June 2020 in central Poland. Antimicrobial susceptibility testing was performed using the disk-diffusion method. *Escherichia coli* (52.2%), *Klebsiella pneumoniae* (13.7%), *Enterococcus faecalis* (9.3%), *E. faecium* (6.2%), and *Proteus mirabilis* (4,3%) were most commonly isolated from urine samples. *E. coli* was significantly more frequent in women (58.6%) (*p* = 0.0089) and in the age group 0–18, while *K. pneumoniae* was more frequent in men (24.4%) (*p* = 0.0119) and in individuals aged 40–60 and >60. Gram-negative species showed resistance to ampicillin. *K. pneumoniae* were resistant to amoxicillin plus clavulanic acid (75.0%), piperacillin plus tazobactam (76.2%), cefotaxime (76.2%), cefuroxime (81.0%), ciprofloxacin (81.0%), and trimethoprim plus sulphamethoxazole (81.0%). Carbapenems were effective against all *E. coli* and *P. mirabilis*. Some *K. pneumoniae* (13.6%) produced metallo-β-lactamases (MBLs). *E. coli* (22.6%), *K. pneumoniae* (81.8%), and all *E. faecium* were multidrug-resistant (MDR). Some *E. coli* (26.2%), *K. pneumoniae* (63.6%), and *P. mirabilis* (14.3%) isolates produced extended-spectrum beta-lactamases (ESBL). Vancomycin-resistant *E. faecium* was also found. This study showed that the possibilities of UTIs therapy using available antibiotics become limited due to the increasing number of antibiotic-resistant uropathogens.

## 1. Introduction

Urinary tract infections (UTIs) belong to the most common bacterial infections in humans [1], accounting for approximately 150–250 million cases globally per year [2]. It is estimated that 40% of women and 12% of men experience minimum one symptomatic UTI during their lifetimes. Moreover, nearly 27–48% of affected women suffer from recurrent UTIs [3,4]. Among all infections acquired in the hospitals, UTIs comprise about 40–50% of bacterial infections that contribute to increased morbidity causing prolonged hospitalization [5,6]. UTIs are also an economic problem because each year worldwide about 150 million people are treated due to UTIs, which costs more than 6 billion dollars [7]. UTIs are clinically divided into complicated or uncomplicated. The complicated UTIs occur in patients with anatomical urinary tract abnormalities or with renal failure, or in patients that use medical devices such as a catheter, with this category of UTIs requiring prolonged therapy. UTIs that occur in patients who have no anatomical urinary tract abnormalities and do not use urinary tract instrumentation are considered uncomplicated [8]. On the basis of clinical presentation and level of severity, UTIs are classified as cystitis (infection of lower UTI), pyelonephritis (infection of upper UTI), or urosepsis (symptomatic UTIs) [9]. The presence of numerous bacterial cells in the urine (≥10^5^ CFU/mL) without the clinical symptoms is called asymptomatic bacteriuria (ABU) [10]. UTIs are mainly caused by *E. coli*, which are responsible for 70–95% of UTI cases [11]. Other etiological agents of UTIs are *Klebsiella pneumoniae* (about 7%), *Proteus mirabilis* (about 5%), *Staphylococcus aureus*, *S. saprophyticus*, and *Enterococcus faecalis* [8]. For treatment of UTIs in all European countries, the following antibacterials are recommended: nitrofurantoin, fosfomycin trometamol, and trimethoprim plus sulfamethoxazole (SXT) [12]. Nitrofurantoin and fosfomycin trometamol are recommended as first-line therapy for uncomplicated cystitis [5,13]. SXT is not recommended due to the high prevalence of bacterial resistance [12,13]. Ciprofloxacin and levofloxacin play an important role in the treatment of more severe infections, such as septicemia, and thus ciprofloxacin should be considered as an alternative, not as a first-line antibiotic, in the treatment of uncomplicated cystitis [12]. Ciprofloxacin could be used as second-line empiric therapy in the case of mild and moderate pyelonephritis or complicated UTI treatment and as third-line empiric treatment for uncomplicated cystitis. Amoxicillin plus clavulanic acid (AMC) is recommended as first-line-therapy for mild and moderate pyelonephritis or complicated UTI and as alternative empiric therapy for uncomplicated cystitis. In the case of complicated UTI or severe pyelonephritis, amoxicillin with gentamicin or a second-generation cephalosporin with an aminoglycoside are recommended as first-line empiric therapy, and third-generation cephalosporin applied intravenously as alternative empiric therapy [13,14]. Uropathogens change, and their antibiotic resistance has increased over the past years, becoming an important problem worldwide. Risk factors for resistance development and appearance of multidrug-resistant (MDR) pathogens in UTIs include prior use of antimicrobials, usually with broad spectrum; previous hospitalization; urinary system anomalies; catheterization; age; and recurrent UTIs [15,16]. Knowledge of regional differences concerning bacterial pathogens responsible for UTIs and their antibiotic susceptibility is important in empirical therapy and for public health to promote proper use of the existing antibiotics. The choice of antimicrobials for UTI treatment is also based on local resistance profiles of uropathogens.

Therefore, the aim of this study was to determine the prevalence of uropathogens reponsible for UTIs in hospitalized patients and to evaluate their antibiotic resistance patterns in order to define the empirical antibiotic treatment for hospitalized patients in the central region of Poland.

## 2. Results

Positive urinary culture results of 161 patients hospitalized between January and June 2020 in a district hospital were more frequently found for women (72.0%) than men (28.0%). The highest percentage of positive urinary cultures was found in patients of nephrology (32.3%), neurological (24.8%), pediatric (15.5%), and internal (15.5%) wards. On the other hand, in the created age groups (0–18, 19–39, 40–60, and >60) the highest percentage of positive samples (73.3%) was found in patients over 60 years of age (Table 1).

Fifteen bacterial species and two species of *Candida* were isolated from 161 positive urine cultures. *E. coli* belonged to the most common bacterial species isolated from urine cultures (*n* = 84, 52.2%), followed by *Klebsiella pneumoniae* (*n* = 22, 13.7%), *Enterococcus faecalis* (*n* = 15, 9.3%), *E. faecium* (*n* = 10, 6.2%), and *Proteus mirabilis* (*n* = 7, 4,3%). *C. albicans* was detected in 6 (3.7%) urine samples. Other microorganism species responsible for UTIs were found in small numbers (Table 2).

*E. coli* as an etiological agent of UTIs was highly significantly more often isolated from the urine of women (*n* = 68, 58.6%) than men (*n* = 16, 35.5%) (*p* = 0.0089), while *K. pneumoniae* was significantly more frequent in men (*n* = 11, 24.4%) than in women (*n* = 11, 9.5%) (*p* = 0.0119) (Table 3).

*E. coli* was the most frequently isolated in the age group of 0–18 years, and the percentage of urine samples (92%) with *E. coli* in this group was significantly higher than in other age groups (Table 4). A higher percentage of urine samples with *K. pneumoniae* was found in the age group of 40–60 (22.2%) and over 60 years (15.2%) than in younger age groups. *E. faecalis* was not found in the youngest patients (0–18). In contrast, the results of the χ^2^ test did not reveal significant differences in the frequency of *E. faecium* isolation between age groups (Table 4).

The presence of *E. coli* in the urine samples from the pediatric ward (92.0%) was highly significantly more frequent than in the urine sample from nephrology (40.4%) (*p* < 0.0001), neurological (52.5%) (*p* = 0.0011), and internal wards (48.0%) (*p* = 0.0008). The results of the χ^2^ test did not reveal significant differences in the frequency of *E. coli* isolation between pediatric and orthopedic wards (60.0%) (Table 5 and Table S1). *K. pneumoniae* was significantly more often isolated from the nephrology patients (26.9%) than from patients of the neurological ward (7.5%) (*p* = 0.0213), while this pathogen was not isolated from the urine of children and patients of the orthopedics ward. *E. faecalis* was the most frequently isolated bacteria from patients in the orthopedics ward (20.0%), but this percentage of isolation did not significantly differ from the percentage of isolation in the nephrology, neurological, and internal wards. Significant differences in the frequency of *P. mirabilis* and *E. faecium* isolation among wards were not found (Table S1).

All *K. pneumoniae* isolates were resistant to ampicillin, and most of them were resistant to penicillins combined with β-lactamase inhibitors (Table 6). A high percentage of *K. pneumoniae* isolates showed resistance to the second and third generation cephalosporins, and also to ciprofloxacin and trimethoprim plus sulphamethoxazole (SXT). The lowest percentage of *K. pnemoniae* isolates was resistant to aminoglycosides and carbapenems. Above half of *E. coli* isolates were resistant to ampicillin and 34.3% showed resistance to amoxicillin plus clavulanic acid (AMC). About one-third of tested *E. coli* were resistant to cephalosporins and SXT. Resistance to ciprofloxacin and gentamicin was found in 28.6% and 24.6% of isolates, respectively. All *E. coli* were susceptible to carbapenems, and 90.3% were susceptible to amikacin. In the group of investigated *P. mirabilis* isolates, a high percentage of isolates were resistant to ampicillin, ciprofloxacin, SXT, and AMC. Contrastingly, all *P. mirabilis* isolates were susceptible to carbapenems (Table 6).

All *E. faecium* isolates were resistant to ampicillin, imipenem, ciprofloxacin, and SXT, and 40.0% showed resistance to gentamicin and vancomycin, while among *E. faecalis*, resistance to vancomycin and imipenem was not observed (Table 7).

Among isolated uropathogens, ESBL-positive isolates were found including *E. coli* (*n* = 22, 26.2%), *K. pneumoniae* (*n* = 14, 63.6%), and *P. mirabilis* (*n* = 1, 14.3%) (Table 8). Above 30% of *E. coli* isolates from patients hospitalized in internal, orthopedics, and nephrology wards were ESBL-positive. The highest percentage of ESBL-positive *K. pneumoniae* (85.7%) was found in the nephrology ward.

Among *K. pneumoniae* isolates, two from internal and one from nephrology wards produced metallo-β-lactamases (MBLs) (Table 9). In the *E. coli* group, 19 (22.6%) MDR isolates were found (Table 9). Among them, two isolates from internal ward patients showed resistance to five antimicrobial agent classes: β-lactams (penicillins, penicillins with β-lactamases inhibitors, cephalosporins), aminoglycosides, fluoroquinolones, nitrofurans, and sulfonamides. Resistance to four antimicrobial agent classes was observed in seven *E. coli* isolates, whereas other MDR isolates were resistant to three antimicrobial groups. Most *K. pneumoniae* isolates (*n* = 18, 81.8%) were MDR (Table 9). Among them, 38.8% (*n* = 7) were resistant to four antimicrobial agent classes; the remainder of isolates were resistant to drugs belonging to three different chemical groups.

All *E. faecium* isolates (*n* = 10) were MDR, including four that showed resistance to vancomycin. All *E. faecium* were resistant to ampicillin, imipenem, ciprofloxacin, and SXT. MDR strains (33.3%) were also found among *E. faecalis* (Table 10).

## 3. Discussion

In our study, we showed that UTIs in patients hospitalized between January and June 2020 in a district hospital in Poland were more frequently found in women (72.0%). Our results are consistent with the other authors’ studies showing that 62.6–86.9% of UTI cases were female [17,18]. Higher prevalence of UTIs among women is due to many different factors that predispose women to this type of infection [19]. This study included different age groups of patients, and it was found that most UTI cases occurred in patients over 60 years of age (72.0%). Our results were similar to those obtained by Woldemariam et al. [20], who showed that the highest percentage of patients with asymptomatic and symptomatic UTI occurred in the age group of 56 years and above.

UTIs are caused mainly by Gram-negative bacteria, with *E. coli* being the most common of them, being responsible for 70–95% of UTI cases [11]. Other Gram-negative etiological agents of UTIs are *Klebsiella* spp., *Enterobacter* spp., and *Proteus* spp. [18]. Potential Gram-positive human uropathogens include *Enterococcus* spp. and *Staphylococcus* spp. [21]. In our study, in positive urinary cultures from 161 hospitalized patients, *E. coli* was a predominant isolate, and its prevalence was 52.2%. Similar results were obtained by Beksac et al. [22] and Demir and Kazanasmaz [18], who detected *E. coli* in 56.7% and 58.9% of the patients, respectively. In this study, the second frequently isolated bacteria were *K. pneumoniae* rods (13.7%), which is in accordance with the results reported by other authors [17,18,23]. In our study, *E. faecalis* (9.3%) and *E. faecium* (6.2%) were less commonly isolated from positive urinary cultures, similarly as reported by Mahony [21], Hrbacek [24], and Woldemariam [20]. Our study showed that *E. coli* was significantly more often isolated from women. Mouanga Ndzime et al. [25] also found that UTI caused by *E. coli* mainly affected women, which may be related to the proximity of the urethra, anus, and vagina in women. In this study, *K. pneumoniae* was significantly more often isolated from men. UTIs caused by *K. pneumoniae* likely depend more on host factors, such as urine flow impairment, e.g., the presence of vesico-ureteral reflux or strictures at the uretero-vesical junction, urinary tract malformation, neurogenic bladder, or benign prostate hyperplasia, than from virulence factors of this opportunistic pathogen [26].

We also found that *E. coli* was significantly more often isolated in the age group of 0–18, while *K. pneumoniae* was significantly more frequent in older persons (40–60 and over 60). This is in agreement with the results obtained by Mouanga Ndzime et al. [25]. These two groups of patients (young and older patients) are more susceptible to urinary tract infection due to the decreased efficiency of the immune system, and in cases of older patients, also impaired function of the urinary tract. Gołębiowska et al. [26] did not find an association between the presence of any investigated genes encoding virulence factors in *K. pneumoniae* and the development of upper UTIs. These authors suggest that UTIs caused by *K. pneumoniae* depend on the patient’s health status and are associated with disturbances in the functioning of the urinary system, which is more often observed in older patients. The global spread of MDR microorganisms has led to an increase in UTIs in humans that are difficult to treat [21]. Ampicillin is one of antibiotics most commonly used in empirical treatment in many parts of the world [18]. In our research, all *K. pneumoniae* isolates, more than half of *E. coli* isolates (53.4%), and the majority of *P. mirabilis* isolates (83.3%) were resistant to ampicillin. High resistance rates of uropathogens to ampicillin have been reported in many parts of the world [11,18,20,25]. This is why the use of ampicillin in empirical therapy of UTI as a single agent is neither appropriate nor recommended. Amoxicillin-clavulanic acid was recommended as an alternative empiric therapy for uncomplicated cystitis or a first-line therapy for moderate pyelonephritis [14]. In this study, more than one-third of the *E. coli* isolates were resistant to a combination of amoxicillin-clavulanic acid (34.3%), and higher resistance rates were observed in cases of *K. pneumoniae* (75.0%) and *P. mirabilis* (60.0%). Resistance to amoxicillin-clavulanic acid was higher in Turkey because 63.1% of *E. coli* isolates and over 80% of *K. pneumoniae* isolates from inpatients were resistant [18]. Earlier research conducted in France showed lower rates of resistance to amoxicillin-clavulanic acid ranging between 25 and 35% [27], which indicates an increase in occurrence of isolates producing β-lactamase enzymes that are not inhibited by clavulanic acid. Resistance to amoxicillin-clavulanic acid is regionally variable. The percentage of *E. coli* resistant to amoxicillin-clavulanic acid isolated from UTI in patients hospitalized in England was 30% [28]. High level of resistance to the combination of these two agents was obtained among *E. coli* isolates from children hospitalized in 2015–2016 in Nepal (48%) [29] and among *E. coli* isolated from patients hospitalized in different hospitals in Jordan (83%) [30]. In this study, a high percentage of *K. pneumoniae* isolates also showed resistance to piperacillin-tazobactam (76.2%), which was higher than that observed in Turkey (45.4%) [18]. In our study, most of *K. pneumoniae* isolates showed resistance to cephalosporins 2nd G (cefuroxime—81.0%) and 3rd G (cefotaxime—76.2%), while these antibiotics were more efficient against *E. coli* and *P. mirabilis* because about 67–68% of *E. coli* and over 83% of *P. mirabilis* isolates were sensitive to them. In contrast, high percentages of isolates belonging to these species that were resistant to cephalosporins (62.5–100%) were obtained in Turkey [18]. In our study, high resistance of *K. pneumoniae* to cefuroxime and cefotaxime suggests that these antibiotics should not be preferred in the empirical treatment of UTIs in this region of Poland. Antibiotics that are recommended for treatment of acute uncomplicated pyelonephritis, complicated UTIs, and urosepsis include carbapenems (ertapenem, imipenem, meropenem) [13]. Carbapenems are also used for the treatment of the infections caused by ESBL-producing bacteria [31]. In our research, all *E. coli* and *P. mirabilis* were sensitive to carbapenems, while three strains isolates (13.6%) of *K. pneumoniae* produced MBLs and showed resistance to this antibiotic group. The emergence of carbapenem-resistant uropathogens makes treating UTIs increasingly challenging. In recent years, carbapenem resistance due to MBL production has been increasingly reported among clinical isolates in the world [32]. Fluoroquinolones are recommended for empirical oral antimicrobial therapy in uncomplicated pyelonephritis [13], and among them ciprofloxacin is the most commonly prescribed because it is available in oral and intravenous preparations. Resistance of uropathogens to fluoroquinolones was reported from different countries, and level of resistance is significant, which is probably related to the excessive use of these antibiotics. In our study, a high percentage of *K. pneumoniae* (81.0%) and *P. mirabilis* (83.3%) showed resistance to ciprofloxacin. On the other hand, among *E. coli* isolates, a lower percentage (28.6%) was resistant to this antibiotic, which is in line with the results of earlier study conducted in Poland, in which resistance to fluoroquinolones was found in about 30% of *E. coli* [33]. Levels of resistance to aminoglycosides (amikacin—9.7% and gentamicin—24.6%) of *E. coli* in our study was similar to that obtained in Turkey, but in our study, the percentage of *K. pneumoniae* and *P. mirabilis* resistant to gentamicin was lower (19.0% and 40.0%, respectively) than in Turkey (41.6% and 50%, respectively) [18]. Activity of amikacin, which is the most active antibiotic among the aminoglycosides, has been preserved against most *E. coli* isolates (above 90% isolates) in our research. In contrast, in case of *K. pneumoniae* and *P. mirabilis*, the activity of this antibiotic was decreased, and resistance rates ranged between 19.0% and 33.3%. Reduced effectiveness of the aminoglycosides can be explained by their frequent administration. SXT is another widely used first-line antimicrobial in the treatment of uncomplicated cystitis, although in many countries, increasing resistance to this drug was observed. A majority of studies show resistance at or above the accepted level of 20%, and thus STX should not be used in empiric treatment [12]. In our study, the resistance rates of uropathogens to SXT ranged between 30.1% in the case of *E. coli* and 81.0% in the case of *K. pneumoniae*. These results indicate that in this region of Poland, SXT should not be used in empiric UTI therapy due to high frequency of uropathogens resistant to this antimicrobial. Nitrofurantoin, according to the European Association of Urology guidelines, is recommended for treatment of uncomplicated cystitis as first-line empiric therapy [13]. A previous study conducted in Romania, Poland, and France showed that the percentage of nitrofurantoin-resistant *E. coli* isolated from outpatients and hospitalized patients ranged from 3 to 3.8% [11,34,35]. The results of the present study revealed increased resistance to nitrofurantoin because the percentage of isolates resistant to this drug was 7.8%. This level of resistance is comparable with that obtained in England (4.6–6.9%) [25] or in Bosnia and Herzegovina (8.23%) [36]. In our previous research, the resistance rate to nitrofurantoin was equal to 3.5% [11]. Despite the increase in the rate of resistance to nitrofurantoin that was more than twice that that in the same region of Poland investigated earlier, nitrofurantoin still remains the drug of choice for treatment of uncomplicated cystitis in this region. Additional characteristics, such as high efficiency, cost effectiveness, and weak adverse environmental impact, justify the use of nitrofurantoin in treatment in uncomplicated UTIs.

In the last few years, *E. faecium* partially replaced *E. faecalis* as a cause of hospital-associated infections [37]. This is a result of a number of mechanisms of natural resistance to antibiotics shown by this microorganism. Moreover, this species is able to acquire resistance by mutations or incorporation of genes located on mobile genetic elements such as plasmids, transposons, or integrons. In this study, all *E. faecium* isolates were MDR, including resistance to ampicillin, imipenem, ciprofloxacin, and SXT, and 40.0% of isolates showed resistance to glycopeptides (VRE—vancomycin-resistant *Enterococcus*) and aminoglycosides (gentamicin). Resistance of *Enterococcus* to glycopeptides has significantly increased over the last years, especially in Bulgaria, Croatia, Denmark, Hungary, Ireland, Italy, Slovakia, and the United Kingdom. The percentage of VRE is variable in different countries, and in Ireland reached 45.5% (European Centre for Disease Prevention and Control Report, 2015). The percentage of VRE isolates in Poland ranges between 8 and 19% [38], while in our research it was equal to 40.0%. This diversity in VRE epidemiology reflects differences in antibiotic and infection control policies in different countries of Europe. There is increased prevalence and dissemination worldwide of *Enterococcus* spp. MDR causes the necessity of searching for new treatment strategies, including combined therapy. The emergence and spread of multidrug-resistant organisms (MDROs) is a global public health threat [39]. Multidrug resistance has been recognized as the consequence of inappropriate use of antibiotics; in particular, a widespread use of cephalosporins and quinolones in humans and animals has been associated with emergence of resistance to these and other antibiotics [20,21]. In our study, we found that 33.0% of uropathogens showed resistance to at least three drug classes. In a group of *E. coli* isolates, 22.6% were MDR, including 52.6% resistant to three groups of antibiotics; the remaining isolates were resistant to four (36.8%) or five (10.5%) antibiotic groups. The study conducted earlier in different parts of Europe (the Netherlands, Germany, and Belgium) showed that 17.5% of *E. coli* isolates were MDR [40]. Similar results as obtained in the present study were reported by Guzmán et al. [41] in Venezuela, where MDR *E. coli* amounted 25.2%. A higher percentage of MDR uropathogenic *E. coli* (63%) was found in Mexico among isolates from community- and hospital-acquired UTIs [42], as well as in Iran (68% and 61% from inpatients and outpatients, respectively) [43]. In contrast, recent reports from India showed an alarming rise in the incidence of multidrug resistance (i.e., simultaneous resistance to aminoglycosides, cephalosporins, fluoroquinolones) among the uropathogenic *E. coli* isolates that varied from 76.51 to 100% [44].

In this study, MDR *E. coli* isolates showed simultaneous resistance to β-lactams, aminoglycosides, fluoroquinolones, nitrofurans, and sulfonamides. Multidrug resistance and its rapid dissemination between *E. coli* by plasmid-encoded conjugation system, which has been considered one of the most important mechanisms for the horizontal transfer of multidrug resistance, complicate therapeutic strategies and threaten public health [44]. In our study, 26.2% of *E. coli* isolates were ESBL producers, about six times more than in previous studies (4.4%) of uropathogenic *E. coli* isolates (2007–2008) from the same hospital [45]. Hrbacek et al. [24] showed that between 2011 and 2019 in a Department of Urology of tertiary referral centers in Central Europe, 8.7% of *E. coli* strains produced ESBL. In the USA in 2017, prevalence of ESBL phenotypes of *E. coli* was 15.7%, and ESBL isolates showed co-resistance to many oral agents [46]. In our study, among *E. coli* ESBL-positive isolates, 72.7% multidrug resistance was observed. We also found that the ESBL-producing isolates showed high frequency (68.7%) of simultaneous resistance to fluoroquinolones and SXT, and 50% showed simultaneous resistance to fluoroquinolones, SXT, and aminoglycosides. In the case of ESBL isolates showing co-resistance to many oral agents, therapeutic options are limited to reserve antibiotics such as carbapenems [47]. In our study, most *K. pneumoniae* isolates (81.8%) were MDR. Moreover, 63.6% of *K. pneumoniae* isolates were ESBL producers, and three isolates (13.6%) produced MBLs. Class B MBLs have a broad substrate spectrum and can catalyze the hydrolysis of all β-lactam antibiotics with the exception of monobactams [48]. In our study, two *K. pneumoniae* MBL-positive isolates, in addition to resistance to β-lactam antibiotics including ertapenem, imipenem, and meropenem, were also resistant to amikacin, ciprofloxacin, and SXT. The study conducted in the Czech Republic showed that in 2011–2019, between 15.4% and 52.3% of *Klebsiella* spp. isolates produced ESBL, while no carbapenem-resistant isolates were found [24]. Treatment options for patients infected with *K. pneumoniae*-producing carbapenemases are very few and are often limited to administration of multiple antibiotic therapies and to colistin [49].

## 4. Materials and Methods

Positive urinary culture results of the 161 hospitalized patients with the diagnosis of UTIs in a district hospital in central Poland were retrospectively evaluated. After examination of the hospital laboratory records, the age, gender, and culture results of the patients as well as the susceptibility and resistance to antibiotics were evaluated. The patients were divided into 4 age groups: 0–18, 19–39, 40–60 and >60 years. These patients showed symptoms including fever, dysuria, and/or loss of bladder control, and/or lower back pain, and/or cloudy or foul-smelling urine. Urine cultures were performed at the microbiological laboratory of the hospital between January and June 2020. Patients with a positive culture, i.e., ≥10^5^ colony forming units per milliliter (CFU/mL), were included in this study. The patients were hospitalized in nephrology, internal, surgery, pediatric, neurological, intensive care, orthopedics, or gynecology units, or a dialysis station.

### 4.1. Isolation and Identification of Uropathogens

Clean catch midstream urine samples were collected in the morning to wide mouth sterile plastic containers. The urine samples were analyzed within 2 h of collection time. All patients were well instructed by trained personnel on how to collect clean catch midstream urine. For children, urine samples were collected using a sterile adhesive collection bag under the supervision of trained personnel. Patients agreed to collect material for diagnostic tests. The urine samples were inoculated onto 5% sheep blood agar and MacConkey and Sabouraud dextrose agar (SDA) (Oxoid, Basingstoke Hampshire, UK) using sterile calibrated loops (10 µL). The inoculations were incubated aerobically at 37 °C for 24 h, while cultures on SDA were incubated at 36 °C for 24–48 h for detection of candiduria. A specimen was considered positive for UTI if a single bacterial species reached a concentration of ≥10^5^ CFU/mL, and significant candiduria was determined as urine culture growth ≥10^4^ CFU/mL. Identification of bacterial isolates was carried out using colony morphology, Gram’s staining, and finally by VITEK 2 Compact System. For the identification of bacteria by VITEK, we used Gram-negative ID cards (GN Reference 21 341) and Gram-positive ID cards (GP Reference 21 342).

### 4.2. Antimicrobial Susceptibility Testing

Antimicrobial susceptibility testing was performed using the disk-diffusion method on Mueller–Hinton agar (bioMérieux, Marcy l’Etoile, France), using antibiotic disks (Oxoid, Basingstoke Hampshire, UK), according to the guidelines of the European Committee on Antimicrobial Susceptibility Testing (EUCAST 2020). The isolates were tested against penicillins: ampicillin (AMP, 10 µg), amoxicillin plus clavulanic acid (AMC, 20 µg + 10 µg), piperacillin plus tazobactam (TZP, 30 µg + 6 µg); cephalosporins: cefuroxime (CXM, 30 µg), cefotaxime (CTX, 5 μg); carbapenems: ertapenem (ETP, 10 µg), imipenem (IMP, 10 μg), meropenem (MEM, 10 µg); fluoroquinolones: ciprofloxacin (CIP, 5 µg); aminoglycosides: amikacin (AMK, 30 μg), gentamicin (GM, 10 μg); other drugs: trimethoprim plus sulfamethoxazole (SXT, 1.25 µg + 23.75 µg), nitrofurantoin (F, 300 µg). Quality control was accessed using *E. coli* ATCC 25922 and *P. aeruginosa* ATCC 27853.

MIC (minimal inhibitory concentration) tests were performed to determine the susceptibility of staphylococci to vancomycin. *S. aureus* ATCC 29213 was used as a control strain. The susceptibility of *Enterococcus* spp. to vancomycin (VA, 30 µg) was performed using the disk-diffusion method and *E. faecalis* ATCC 29212 as control strain. ESBL production was detected using the double disk synergy test [50]. *E. coli* ATCC 35218 was used as control strain. The combined disc assay using imipenem and imipenem/ethylenediaminetetraacetate discs was used to confirm production of MBLs by isolates showing resistance to imipenem [51]. Isolates resistant to 3 or more classes of antimicrobial agents were considered multidrug-resistant (MDR).

### 4.3. Data Analyses

Chi-squared statistics in Statistix 11.0 (Analytical Software, Tallahassee, FL, USA) was used for testing significance of differences between pairs of proportions (percentages) of isolates and the source of isolation (hospital ward), gender, and age. Differences at *p* ≤ 0.05 were regarded as significant, while at *p* ≤ 0.01 as highly significant.

### 4.4. Ethical Approval

For this type of study, formal consent is not required because this article does not contain any studies with human participants or animals performed by any of the authors. Patient consent was waived due to the retrospective design of the study. Patients and caregivers gave their written consent to use their personal data at the admission into the hospital. Anonymity of patients was guaranteed during the whole process of data analysis and reporting of the results. An official permission letter was also obtained from the Director of the Our Lady of Perpetual Help Hospital in Wołomin to use the data for analysis.

## 5. Conclusions

Our study confirmed that *E. coli* is the main uropathogen in inpatients, although *K. pneumoniae* was also frequently isolated from UTI cases. UTIs were more frequent in women and in older persons. Our results also showed that *E. coli* was significantly more often isolated from women and persons from the youngest age group, while *K. pneumoniae* was isolated more from men and older persons. These uropathogens showed high resistance rates to ampicillin. Cephalosporins of 2nd and 3rd G showed high antibacterial activity against most *E. coli* and *P. mirabilis*, while most of *K. pneumoniae* strains were resistant to this group of antibiotics. Carbapenems were effective against all *E. coli* and *P. mirabilis* and most *K. pneumoniae*. Ciprofloxacin should not be used in the empirical therapy of UTIs caused by *K. pneumoniae* and *P. mirabilis* because most strains belonging to this species were resistant. Amikacin was active against most *E. coli*, while resistance rates to this aminoglycoside in *K. pneumoniae* and *P. mirabilis* isolates were higher. In the region of Poland under assessment, SXT should not be used in empiric UTI therapy due to high frequency of uropathogens resistant to this antimicrobial, while nitrofurantoin still remains the drug of choice for treatment of uncomplicated cystitis caused by *E. coli*. This study also showed that many uropathogens were MDR, especially *K. pneumoniae*, and among them, many were ESBL producers; few *K. pneumoniae* produced MBLs. UTIs caused by bacteria that produce ESBL and MBLs are very difficult to treat and can often lead to treatment failure.

## Figures and Tables

**Table 1:** Positive urine cultures according to hospital ward, age, and gender of patients.

Characteristics	*n*	%
Hospital Ward		
Nephrology	52	32.3
Neurological	40	24.8
Pediatric	25	15.5
Internal	25	15.5
Intensive care	5	3.1
Orthopedics	5	3.1
Gynecology	4	2.5
Surgery	3	1.9
Dialysis station	2	1.2
Age		
0–18	25	15.5
19–39	9	5.6
40–60	9	5.6
>60	118	73.3
Gender		
Female	116	72.0
Male	45	28.0

**Table 2:** Distribution of microorganisms isolated from urine samples.

Microorganisms	*n*	%
*Escherichia coli*	84	52.2
*Klebsiella pneumoniae*	22	13.7
*Enterococcus faecalis*	15	9.3
*Enterococcus faecium*	10	6.2
*Proteus mirabilis*	7	4.3
*Candida albicans*	6	3.7
*Enterobacter cloacae*	3	1.9
*Streptococcus agalactiae*	2	1.2
*Pseudomonas aeruginosa*	2	1.2
*Staphylococcus haemolyticus*	2	1.2
*Candida glabrata*	1	0.6
*Morganella morganii*	1	0.6
*Staphylococcus aureus*	1	0.6
*Acinetobacter baumanii*	1	0.6
*Providencia stuarti*	1	0.6
*Klebsiella oxytoca*	1	0.6
*Cupriavidus pauculus*	1	0.6
*Streptococcus* spp.	1	0.6

**Table 3:** The occurrence of the most common uropathogens in women and men hospitalized in different hospital wards.

Uropathogens	Female (*n* = 116)	Male (*n* = 45)	
	Occurrence of Uropathogens		
	*n* (%)	*n* (%)	*p*-Value
*E. coli*	68 (58.6)	16 (35.5)	0.0089 **
*K. pneumoniae*	11 (9.5)	11 (24.4)	0.0119 *
*P. mirabilis*	5 (4.3)	2 (4.4)	-
*E. faecalis*	11 (9.5)	4 (8.8)	-
*E. faecium*	6 (5.2)	4 (8.8)	0.3434

* *p* < 0.05, ** *p* < 0.01 (χ^2^ test).

**Table 4:** The prevalence of major uropathogens in different age groups of hospitalized patients.

	Age Groups				*p*-Value		
Uropathogens	0–18 A (*n* = 25)	19–39 B (*n* = 9)	40–60 C (*n* = 9)	>60 D (*n* = 118)	Comparison of:		
	Occurrence of Uropathogenes, *n* (%)				A and B	A and C	A and D
*E. coli*	23 (92.0)	5 (55.5)	3 (33.3)	53 (44.9)	0.0164 *	0.0004 **	<0.0001 **
*K. pneumoniae*	0	1 (11.1)	2 (22.2)	18 (15.2)	0.0973	0.0173 *	0.0393 *
*P. mirabilis*	0	0	0	7 (5.9)	-	-	0.2106
*E. faecalis*	0	2 (22.2)	2 (22.2)	11 (9.3)	0.0173 *	0.0173 *	0.1203
*E. faecium*	1 (4.2)	1 (11.1)	1 (11.1)	7 (5.9)	0.4498	0.4498	0.6950

* *p* < 0.05, ** *p* < 0.01 (χ^2^ test).

**Table 5:** The prevalence of major uropathogens in different hospital wards.

Uropathogens	Hospital Ward				
	Nephrology (*n* = 52)	Pediatric (*n* = 25)	Neurological (*n* = 40)	Internal (*n* = 25)	Orthopedics (*n* = 5)
	Occurrence of Uropathogenes, *n* (%)				
*E. coli*	21 (40.4)	23 (92.0)	21 (52.5)	12 (48.0)	3 (60.0)
*K. pneumoniae*	14 (26.9)	0	3 (7.5)	4 (16.0)	0
*P. mirabilis*	2 (3.8)	0	2 (5.0)	1 (4.0)	0
*E. faecalis*	7 (13.5)	0	3 (7.5)	3 (12.0)	1 (20.0)
*E. faecium*	3 (5.7)	1 (4.0)	3 (7.5)	3 (12.0)	0

**Table 6:** Antimicrobial resistance of Gram-negative bacteria isolated from urine culture of hospitalized patients from January to June 2020.

Antimicrobial Group	Antimicrobials	*E. coli*	*K. pneumoniae*	*P. mirabilis*
		% Resistant Isolates		
Penicillins	Ampicillin	53.4	100	83.3
	AMC	34.3	75.0	60.0
	TZP	13.2	76.2	33.3
Cephalosporins 2nd G	Cefuroxime	32.9	81.0	16.7
Cephalosporins 3rd G	Cefotaxime	31.5	76.2	16.7
Carbapenems	Ertapenemem	0.0	13.6	0.0
	Imipenem	0.0	13.6	0.0
	Meropenemem	0.0	13.6	0.0
Fluoroquinolones	Ciprofloxacin	28.6	81.0	83.3
Aminoglycosides	Amikacin	9.7	19.0	33.3
	Gentamicin	24.6	19.0	40.0
Sulfonamides	SXT	30.1	81.0	66.7
Nitrofurans	Nitrofurantoin	7.8	-	-

AMC—amoxicillin plus clavulanic acid, TZP—piperacillin plus tazobactam, SXT—trimethoprim plus sulphamethoxazole, “-” not tested; the most effective antibiotics marked in red, the least effective antibiotics in green.

**Table 7:** Antimicrobial resistance of Gram-positive bacteria isolated from urine culture of hospitalized patients from January to June 2020.

Antimicrobial Group	Antimicrobials	*E. faecalis*	*E. faecium*
		% Resistant Isolates	
Penicillins	Ampicillin	7.7	100
Carbapenems	Imipenem	0.0	100
Fluoroquinolones	Ciprofloxacin	36.4	100
Aminoglycosides	Gentamicin	38.5	40
Glycopeptides	Vancomycin	0.0	40
Sulfonamides	SXT	46.2	100

SXT—trimethoprim plus sulphamethoxazole; the most effective antibiotics marked in red.

**Table 8:** Occurrence of ESBL-positive uropathogens in patients hospitalized in different hospital wards.

Uropathogens (*n*)	No. (%) of ESBL-Positive Isolates	Hospital Wards (*n*, %)
*E. coli* (84)	22 (26.2)	Nephrology (8, 30.1), neurological (5, 23.8), internal (4, 33.3), pediatric (4, 17.4), orthopedics (1, 33.3)
*K. pneumoniae* (22)	14 (63.6)	Nephrology (12, 85.7), internal (1, 25.0), dialysis station (1, 100)
*P. mirabilis* (7)	1 (14.3)	Nephrology (1, 50.0)

**Table 9:** Multi-drug resistance pattern of Gram-negative bacteria isolated from urine culture of hospitalized patients from January to June 2020.

Combination of Drugs (No. of Antimicrobial Agents Classes)	No. of Isolates	Resistance Mechanism (*n*)	Hospital Wards (*n*)
*E. coli*			
AMP, AMC, GM, SXT (3)	1	-	Neurological
AMP, CXM, CTX, F, GM (3)	1	ESBL	Neurological
AMP, CXM, CTX, AMC, CIP, SXT (3)	2	ESBL (2)	Internal, nephrology
AMP, CXM, CTX, TZP, AMC, GM, CIP (3)	1	ESBL	Internal
AMP, CXM, CTX, AMC, F, SXT (3)	1	ESBL	Pediatric
AMP, CXM, CTX, CIP, SXT (3)	1	ESBL	Internal
AMP, CXM, CTX, TZP, AMC, AK, GM, CIP (3)	1	ESBL	Nephrology
AMP, CXM, CTX, TZP, AMC, AK, GM, SXT (3)	1	ESBL	Neurological
GM, CIP, SXT (3)	1	-	Nephrology
AMP, CXM, TZP, AMC, F, GM, SXT (4)	1	-	Nephrology
AMP, CXM, CTX, AMC, GM, CIP, SXT (4)	3	ESBL (3)	Nephrology (2), pediatric
AMP, CXM, CTX, TZP, AMC, GM, CIP, SXT (4)	2	ESBL (2)	Nephrology, neurological
AMP, CXM, CTX, GM, CIP, SXT (4)	1	ESBL	Pediatric
AMP, CXM, CTX, AMC, F, GM, CIP, SXT (5)	1	ESBL	Internal
AMP, CXM, CTX, TZP, AMC, F, AK, GM, CIP, SXT (5)	1	ESBL	Internal
*K. pnemoniae*			
AMP, CIP, SXT (3)	1	-	Internal
AMP, CXM, CTX, CIP, SXT (3)	1	-	Neurological
AMP, CXM, CTX, TZP, AMC, CIP, SXT (3)	8	ESBL	Nephrology (7), dialysis station (1)
AMP, CXM, CTX, IMP, MEM, ETP, TZP, AMC, CIP, SXT (3)	1	MBL	Nephrology
AMP, CXM, CTX, TZP, AMC, AK, CIP, SXT (4)	2	ESBL	Nephrology
AMP, CXM, CTX, TZP, AMC, GM, CIP, SXT (4)	3	ESBL	Nephrology (2), internal (1)
AMP, CXM, CTX, IMP, MEM, ETP, TZP, AMC, AK, CIP, SXT(4)	1	MBL	Internal
AMP, CXM, CTX, IMP, MEM, ETP, TZP, AMC, AK, GM, CIP, SXT (4)	1	MBL	Internal

AMP—ampicillin, AMC—amoxicillin plus clavulanic acid, TZP—piperacillin plus tazobactam, CXM—cefuroxime, CTX—cefotaxime, ETP—ertapenemem, IMP—imipenem, MEM—meropenemem, CIP—ciprofloxacin, AMK—amikacin, GM—gentamicin, SXT—trimethoprim plus sulphamethoxazole, F—nitrofurantoin.

**Table 10:** Multi-drug resistance pattern of Gram-positive bacteria isolated from urine culture of hospitalized patients from January to June 2020.

Combination of Antibiotics (No. of Antimicrobial Agent Classes)	No. of Isolates	Resistance Mechanism	Hospital Wards (*n*)
*E. faecium*			
AMP, IMP, CIP, SXT (3)	2	-	Neurological
AMP, IMP, CIP, VA, SXT (4)	2	VRE	Nephrology, neurological
AMP, IMP, CIP, GM, SXT (4)	4	-	Internal (2), nephrology, pediatric
AMP, IMP, CIP, F, VA, SXT (5)	1	VRE	Internal
AMP, IMP, CIP, GM, VA, SXT (5)	1	VRE	Nephrology
*E. faecalis*			
GM, CIP, SXT (3)	4	-	Nephrology (2), internal (1), orthopedics (1)
AMP, GM, CIP, SXT (4)	1	-	Internal

AMP—ampicillin, IMP—imipenem, CIP—ciprofloxacin, GM—gentamicin, SXT—trimethoprim plus sulphamethoxazole, F—nitrofurantoin, VA—vancomycin, VRE—vancomycin-resistant *Enterococcus*.

## Data Availability

The data presented in this study are available in the article and in the supplementary material.

## Supplementary Materials

The following are available online at https://www.mdpi.com/article/10.3390/antibiotics10040447/s1, Table S1: Results of analysis using of chi-squared statistics for calculations in order to find significant differences between pairs of proportions (percentages) of isolates and the source of isolation (hospital ward).
