# Supplementary material for: Antibiotic Resistance of Uropathogens Isolated from Patients Hospitalized in District Hospital in Central Poland in 2020
Source: Antibiotics (Basel). 2021 Apr 16;10(4):447. doi: 10.3390/antibiotics10040447 (PMC8071495; doi:10.3390/antibiotics10040447)
Supplement: Supplementary file 1 [file antibiotics-10-00447-s001.zip › antibiotics-1186590-supplementary.pdf]

Table S1. Results of analysis with using of Chi-square statistics for calculations in order to find significant differences between pairs of proportions (percentages) of isolates and the source of isolation (hospital ward).

**Hospital Ward**	*Enterococcus* *faecalis*	*Enterococcus* *faecium*	*E. coli*	*Klebsiella pneumoniae*	*Proteus miriabilis*
	***p* Value**				
Nephrology × Internal	0.9023	0.3652	0.5090	0.2891	x
Nephrology × Nephrology	0.4467	0.7085	0.2171	**0.0213** *	0.8183
Nephrology × Orthopaedics	0.6657	0.5766	0.3906	0.1847	0.6516
Nephrology × Paediatric	0.0608	0.7168	**<0.0001** **	**0.0043****	0.3138
Internal × Nephrology	0.5961	0.5961	0.6971	0.3212	0.8528
Internal × Orthopaedics	0.6367	0.4221	0.6300	0.3449	0.6547
Internal × Paediatric	0.0770	0.3020	**0.0008** **	**0.0390** *	0.3173
Nephrology × Orthopaedics	0.3898	0.5164	0.7698	0.5164	0.6130
Nephrology × Paediatric	0.1501	0.5266	**0.0011** **	0.1501	0.2598
Orthopaedics × Paediatric	**0.0253** *	0.6547	0.0589	x	x

Data with p value of less than 0.05 was regarded as significant, while with p value of less than 0.01 was regarded as highly significant.
